# Impact of COVID-19 second wave on healthcare worker staffing levels

**DOI:** 10.1017/ice.2020.353

**Published:** 2020-07-22

**Authors:** Ala Abuown, Catharine Taube, Louis J. Koizia

**Affiliations:** 1 Imperial College Healthcare NHS Trust, London, United Kingdom; 2 Cutrale Perioperative and Ageing Group, Imperial College London, London, United Kingdom

*To the Editor—*The coronavirus disease 2019 (COVID-19) pandemic prompted mass restructuring of the NHS workforce, the scale of which was unprecedented in its 72-year history. Like many others, Imperial College Healthcare Trust rolled out expanded emergency COVID-19 rotas, with built-in shadow cover in response to the expected high rates of staff absence.^1^ To meet the heavy staffing requirement of these COVID-19 rotas, healthcare workers (HCWs) from nonemergency, surgical, community, allied health, and academic posts were temporarily redeployed.^2^ But with the second peak of COVID-19 predicted in November 2020, during a time of yearly maximal pressure on the NHS, will we be able to maintain safe staffing levels?

To better understand the impact of COVID-19 on staffing levels, we undertook a survey of 167 healthcare workers (HCWs) at St Mary’s Hospital. Overall, 44% reported that they had had symptoms of COVID-19 and had self-isolated at some point over the previous 4 months. Among responders, 18% reported self-isolating while asymptomatic due to a symptomatic member of their household. The median isolation period was 10–14 days, which is in line with Public Health England (PHE) guidance. Moreover, 48% of staff reported living with at least 1 other HCW. Therefore, a positive swab in a singular household, on average, affected 2 HCWs in our surveyed group.

The advent of high-sensitivity antibody test in May 2020 was widely seen as a vital turning point in the COVID-19 response. Many NHS trusts have now rolled out staff-wide antibody testing. As of June 2020, Imperial College Healthcare Trust reported that 25% of staff tested had positive IgG, with other trusts reporting similar rates.^3^

However, antibody testing has yet to produce a tangible impact on staffing. Discussions around the degree and length of immunity a previous infection provides remain largely speculative. Research to address these vital questions is ongoing. Presently, NHS England advises that staff should continue following PHE isolation guidance even if they have a positive antibody test. Thus, for the foreseeable future, individuals who have already been infected and have a positive antibody test will need to isolate if a member of their household becomes symptomatic. Furthermore, they will need to isolate if contacted as part of the ‘test and trace’ strategy despite having recovered from the virus and working on the frontline. As such, the rollout of antibody testing does not stop the domino effect on HCW staff depletion in the event of a second wave.

A second wave will also bring new challenges. Previous pandemics, such as the swine flu pandemic in 2009, have exhibited second waves deadlier than the first. Meeting the demand for HCWs during a winter spike, at which time the NHS is already under tremendous seasonal pressure, will be a mammoth task. We expect HCWs to be extra vigilant for COVID–19 symptoms, potentially increasing the numbers of HCWs self-isolating while awaiting a swab test. The government ‘test and trace’ strategy asks anyone who has had a confirmed interaction with a person who tests positive for COVID-19 to isolate for 14 days. Given the backlog and increasing patient waiting lists as a result of the first wave, there is likely to be resistance to redeployment on the same scale to cope with further waves.

We are faced with the question of how we can be better prepared to staff the second wave. Up to one-quarter of the HCWs at our London trust have had positive antibody tests. We need to have clear guidelines on how we use these data and what they mean for HCWs. Crucially, is there a way we can to avoid the same workforce depletion we experienced during the first wave? We need a clear strategy to maintain safe levels of HCW staffing in a second wave that has the potential to be more complex than the first. These issues need to be addressed by PHE, and we feel that a special set of guidance should be created for frontline HCWs.
